# It’s Just a Distance Thing: Affordances and Decisions in Online Disclosure of Sexual Violence Victimization

**DOI:** 10.1177/08862605241246800

**Published:** 2024-04-16

**Authors:** Marleen Gorissen

**Affiliations:** 1 Vrije Universiteit Amsterdam, The Netherlands; 2 Netherlands Institute for the Study of Crime and Law Enforcement (NSCR), Amsterdam, The Netherlands

**Keywords:** online disinhibition, sexual violence, online disclosure, social media, #MeToo, victim

## Abstract

The Internet offers an alternative context in which personal experiences with sexual violence can be shared. It has been suggested that victims experience lower barriers to disclosing their stories in a digital environment due to an online disinhibition effect and mainly anonymity. However, little is known about the lived experiences of victims who have shared their experiences online regarding these disinhibiting affordances of the Internet. Twenty-three interviews with victims were conducted to understand the digital affordances involved in the online disclosure of sexual victimization. The results suggest that the Internet offers several opportunities (visibility management, asynchronicity, and connectivity) and constraints (lack of non-verbal communication, disclosing online is irreversible, and Internet as a source of triggers) when disclosing sexual violence victimization online. We learn that disclosures are informed by previous experiences and weighed against digital affordances. Victims use multiple platforms or multiple accounts on the same platform and manipulate anonymity and visibility through the settings of online platforms. The Internet offers a potential for informal online peer support. The results have practical implications for victims, clinicians, and support providers for guiding the disclosure process. Furthermore, a re-evaluation and nuance of the online disinhibition theory is suggested. Suggestions for future research are made.

A few months after the #MeToo movement, a national study on sexual harassment and assault in the United States revealed that 81% of women and 43% of men experienced some form of sexual violence victimization (Kearl, 2018). After experiencing sexual violence, individuals deal with their victimization in different ways. One way of coping with victimization and its consequences (either physical, psychological, or material) is to disclose what has happened. Disclosure can be described as sharing information about oneself with others (Greene et al., 2006), for example, with friends or family members (known as informal disclosure) or with a therapist, or the police (known as formal disclosure; Ullman and Filipas, 2001). Sharing experiences of sexual violence can help process the incident emotionally and cognitively, which is related to an improvement in mental and psychological health (Pennebaker et al., 2001; Rime, 1995). Disclosure is considered goal-oriented behavior (Omarzu, 2000). It involves a process of balancing safety (by keeping information to themselves) and a wish to reveal and be seen (by sharing information), in which victims attempt to limit the perceived risks of disclosure (Bachman, 1993; Browne, 1991; Petronio, 2002). The social reactions victims receive after a disclosure determine their willingness to continue to talk about it (Ahrens et al., 2007; Ullman, 2011) and whether a disclosure is beneficial (e.g., Ullman & Peter-Hagene, 2014). Victims of sexual violence are more likely to share their stories with friends or family than to disclose formally (Ahrens et al., 2007). Nevertheless, there is a relatively large group of victims who never disclose their experiences to anyone or wait years to do so (e.g., D. W. Smith et al., 2000). Sexual violence can be considered a stigmatized experience (Kennedy & Prock, 2018). The barriers to disclose can be personal (e.g., feelings of guilt, shame, self-blame, and fear; e.g., Kennedy and Prock, 2018); Ullman (2002) and context-related, such as the sociocultural milieu in which a person is victimized (Ahrens et al., 2010; Tillman et al., 2010). The weighing process to disclose for victims of sexual violence has been well-studied in the *offline* context (e.g., Bachman, 1993; Browne, 1991; Feldman-Summers & Norris, 1984). What this process looks like in the online world is still unknown and therefore the subject of this study.

Numerous international studies indicate that the online world is increasingly becoming a place for victims to share their experiences outside or alongside conventional offline sources. Moors and Webber (2012), for example, argue the Internet offers a place for disclosure when victims are unsuccessful in finding offline help. The offline barriers to disclosure, such as shame and fear of repercussions, may be removed by the changed context and characteristics of the Internet (Andalibi et al., 2016). Where in the offline context, a recipient of disclosure is generally known or at least visible, in the digital context a technology mediates between the discloser and the audience. Online, a recipient can be anyone and posts have a historical archive. A systematic literature review revealed that victims of sexual violence disclose their experiences online to seek or provide support and validation, to document, educate, and unburden, to seek justice, or as a form of activism (Gorissen et al., 2021). It should be noted that the studies on online disclosure of sexual violence victimization mainly focus on those who shared during highly visible hashtag campaigns (such as #MeToo) and on public social media platforms (Gorissen et al., 2021). Salter (2013), Powell (2015), and Fileborn (2017) show that online spaces can function as counter-cultural public spheres in which dominant social, cultural, and legal representations of sexual violence are challenged. Although access and ability to use the Internet to disclose are not universal (e.g., Latina & Docherty, 2014), formal channels of criminal justice can be circumvented: via the Internet, victims can give voice to their own experiences while perpetrators can be “punished” or publicly condemned (Fileborn, 2017). Online spaces or platforms are connected to the Internet and facilitate the generation, sharing, and storage of text, image, and video content (Powell, 2015). Platforms to disclose sexual victimization include social media such as Twitter, Facebook, Instagram, and YouTube and blogs and peer support forums.

Omarzu (2000) states that *offline* disclosure is a curated process in which meticulous decisions are made about whom to disclose, what to disclose, and how to disclose. These decisions are influenced by the context in which the disclosure takes place, the (target) audience, the anticipated reactions to a disclosure (Petronio, 2002), and the motivation or function (e.g., self-expression; Derlega and Grzelak, 1979) to disclose (Derlega & Grzelak, 1979; Omarzu, 2000). To the best of my knowledge is this process largely unknown for *online* disclosure of sexual violence. Each online platform has its architecture, rules, and style that can influence users’ decisions to share certain experiences. The possibilities embedded in the structure of the platforms limit particular modes of expression or articulation (Gillespie, 2010) and give priority to specific forms of social participation (Gibbs et al., 2015).

Previous research (e.g., Andalibi et al., 2016; Bogen et al., 2018; Fawcett & Shrestha, 2016) proposed that the Internet can lower the threshold for sharing personal experiences due to anonymity and accessibility (L. Smith, 2010) and referred to the online disinhibition theory by Suler (2004). This theory describes the effect the Internet can have on lowering someone’s (behavioral) restraints (Suler, 2004). On the Internet, people more openly express (personal) information and say and do things that they would not normally say or do in the physical world, because they are less concerned about self-presentation and judgment of others (Joinson, 1998; Suler, 2004). Elements of the Internet can remove psychological barriers (such as shame and guilt) to disclose. These elements will be discussed in more detail below.

## The Online Disinhibition Theory

Suler (2004) describes six dimensions that influence the reduction of behavioral restraints on the Internet, including (dissociative) anonymity, invisibility, and asynchronicity. The remaining three dimensions, minimization of status and authority, dissociative imagination, and solipsistic introjection, appear to be less relevant in this context. Studies on online disclosure of sexual victimization often fail to mention any of the dimensions described by Suler (2004) or merely present one or more of the first three as an underlying assumption. The online disinhibition theory presents the opportunities the Internet affords victims.

### Anonymity

Suler (2004) describes dissociative anonymity, where online actions can be separated from someone’s identity in the offline world and can even lead to the construction of a compartmentalized online self. Victims disclosing their names or identities on the Internet can have serious consequences in the offline world—anonymity can reduce victims’ vulnerability during and after disclosure (O’Neill, 2018).

### Invisibility

Next to concealing one’s identity, the fact that people cannot see each other online (invisibility) contributes toward peoples’ appetite and courage for disclosing things they would not share in the physical world (Suler, 2004). Although people are not always anonymous online, they are nevertheless invisible to their audience (Suler, 2004).

### Asynchronicity

Asynchronicity is considered the third threshold-lowering element of the Internet that may influence online disclosure of sexual violence. Online, one can take time to construct a message—a delay—because no immediate response is required (Suler, 2004), increasing the ease with which victims disclose. After posting an emotional and personal message (such as a disclosure of sexual victimization), the computer can simply be turned off.

Concluding, the Internet could remove (some of) the barriers to disclosing sexual violence and provide an alternative context to disclose sexual victimization.

In studies on online disclosure of sexual violence (e.g., Andalibi et al., 2016; Bogen et al., 2018; Fawcett & Shrestha, 2016), the possibility of remaining anonymous, in contrast to offline forms of sharing, is the most frequently proposed explanation for the online facilitation of disclosure. The theory offers further insight into how victims who have decided to disclose their experiences use the affordances of the Internet to their advantage. Affordances are perceived abilities by users that “enable or constrain potential behavioral actions in a particular context” (Evans et al., 2017, p. 36). In other words, affordances are the kind of uses an object affords. Moreno and D’Angelo (2019) present several affordances of the Internet, namely functional, social, cognitive, emotional, and identity affordances. Zooming in on the latter, identity affordances are “opportunities on social media platforms for identity development and portrayal” (Moreno & D’Angelo, 2019, para. 7). For example, on the Internet users can create separate profiles to highlight or conceal certain aspects of their identity.

The relationship between the affordances of the Internet and online disclosure of sexual violence has been suggested in numerous studies (e.g., Andalibi et al., 2016; Deal et al., 2020; O’Neill, 2018). These studies used the *content* of the online post to make statements about how features of specific social media platforms had been used. Particular online affordances are highlighted on particular platforms, for example, anonymity on Reddit (Andalibi et al., 2016) or social affordances in Twitter posts (Deal et al., 2020). Illustrating, Andalibi et al. (2016) compared the content of posts that were shared using pseudonyms or so-called “throwaway accounts,” two variations of anonymous accounts. This comparison, however, informs little about the underlying rationale for choosing a form of anonymous disclosure (or non-anonymous options) and other affordances of the Internet that were weighed.

Concluding, it has been theorized that the Internet lowers the threshold for the sharing of sexual victimization, but empirical evaluation of the relationship between digital affordances and online disclosure is lacking. Even less is known about the lived experiences of victims who disclose sexual victimization online. Whether or not victims indeed experience online disinhibition and which affordances offer opportunities or constraints when disclosing on the Internet needs further evaluation (Bogen et al., 2021, p. 3).

## The Current Study

This study focuses on the lived experiences of victims to understand their online disclosure process, the decisions they make, and the online affordances that offer both opportunities and constraints for online disclosure of sexual violence. In light of the research tradition on online disclosure of sexual victimization in which no infrequent reference is made to the online disinhibition theory as an explanatory mechanism, this theory was used as a conceptual framework. This means that the findings will be placed against the central propositions of the online disinhibition theory by Suler (2004), considering how it does or does *not* fit online disclosure of sexual violence. In other words, how the Internet may offer both opportunities and constraints. To make lasting claims about digital affordances in relation to online disclosure of sexual victimization, affordances of the Internet rather than features of specific online platforms will be examined. In the Results section of this article, particular platforms will sometimes be mentioned to illustrate digital affordances.

Insight into online affordances can have implications for clinicians in terms of assistance to victims in the procedure. The Internet may offer victims affordances that act as opportunities, such as anonymity and invisibility to share their experiences that the *offline* world does not or not sufficiently afford them. Knowing when victims feel they can safely and meaningfully disclose may inform considerations victims make upon sharing their victimization. This underlines the need to explore the decisions and choices to guide victims toward a balanced view of both the opportunities and constraints of the Internet.

## Methods

The study aims to gain insight into the online affordances of the Internet that offer both opportunities and constraints. Interviews with victims of sexual victimization who disclosed online offer a rich understanding of how affordances of the Internet are weighed. The members of the Ethics Committee of the Faculty of Law and Criminological research of the Vrije Universiteit Amsterdam advised positively on the execution of the study and identified no ethical concerns.

Respondents were victims of sexual violence who shared their stories publicly online and were willing to discuss the online disclosure of their experience(s). Sexual violence is used as a general term to describe a broad spectrum of unwanted sexual experiences, physical or verbal, attempted and completed acts taking place without the consent of the individual and/or by the use of force.

### Recruitment

Respondents had to have experienced sexual violence, have shared their stories online on a public platform, and be willing to talk about the motivations for the online disclosure of their stories. Respondents were selected from a database of Dutch public social media messages on sexual victimization (for more information on the composition of this database, see Gorissen et al. (2023)]. The complete database consisted of 599,591 posts, of which 100,000 posts were randomly selected and manually annotated as either disclosures of personal stories of sexual violence victimization or nondisclosures. A total of 288 unique disclosure posts formed the sample of which potential respondents were identified. The researcher created profiles on Twitter, Facebook, and Instagram to contact potential respondents on which the researcher identified herself with her first name, a photo, and a short biography. This biography expressed that the online profile was created to conduct scientific research. Potential respondents were contacted when their privacy settings allowed the researcher to send a private message through the platform they had used to disclose their sexual victimization. Private messaging allows for the communication to remain confidential and accessible only to the concerned parties. Recruitment was ongoing during the interviews. In the periods July 17, 2018 to August 22, 2018, and January 1, 2019 to June 7, 2019, 42 respondents were contacted through this method. Furthermore, a call for participation was made in a newsletter of an influential peer support provider for victims of sexual violence, allowing for the self-selection of potential respondents.

In the first contact, the purpose and aim of the study were explained. The message emphasized that the interview would focus on the experiences with and motivations for online disclosure. It was stressed that the victimization itself did not have to be discussed. If the initial message elicited no reply, a follow-up was sent after 2 weeks. Upon positive contact, communication generally shifted to e-mail or telephone. Questions, if any, were answered and an appointment was made for the interview. Upon non-response or when the respondent declined contact, communication was deleted and this information was added to the database with a dichotomous variable (participating interview: Y/N). In both the contact with victims and the Result section of this paper the term “victim” was avoided, since participants of the study might not have identified as such.

Of the 42 respondents who were approached via social media, 18 participated in the study. Eight respondents gave a reason for opting out. These reasons were health problems preventing participation and lack of time/unavailability. The newsletter elicited an additional seven people interested in taking part, of which ultimately, five participated. The other two did not meet the inclusion criteria. The 23 interviewed offered rich and nuanced data and therefore formed the final sample. Additionally, during the last interviews no new themes were found. The participant group consisted of 21 women, 1 man, and 1 nonbinary person. Other demographic information such as age, ethnicity, and socioeconomic status were not captured during the data collection. The nonbinary pronoun “they” will be used to refer to (singular) respondents to prevent misgendering.

### Procedure

In the Netherlands, face-to-face interviews are common due to the country’s relatively small geographical size and efficient transportation infrastructure. All but one interview took place in person, at a location of the respondent’s choosing such as at their homes or in a public place (café or library), to prioritize the respondent’s comfort and safety. One respondent did not feel comfortable talking in person and therefore answered questions via e-mail, to which the interviewer was able to respond and ask follow-up questions.

Before proceeding with the interview, the purpose of the study was once again explained. Respondents filled out an informed consent form, warranting the respondents’ voluntariness, confidentiality, and anonymity and providing information about data storage and data protection. It was explained that the conversation could be terminated at any time. Finally, permission was asked for audio-recording the conversation on a pin-protected recorder. All respondents agreed and signed the informed consent.

The interviews had a semi-open structure. A topic list based on previous research on online disclosure of sexual violence (Van den Berg & Gorissen, 2020) was used. Open-ended questions were asked about, among other things, the motivation to disclose experiences with sexual victimization and the considerations before disclosing regarding the platform choice, target audience, content of the post, and identity revelation (e.g., “What considerations did you make regarding the content of the message?). Exploratory and clarifying questions were asked, in which respondents were invited to reflect on the decisions they had made. These questions were not specifically related to the concepts of the online disinhibition theory to avoid steering the answers.

The topic list was used to guide the conversation. The aim was to offer the respondents the opportunity to talk about things that were important to them. The interviews were all concluded by asking whether there were subjects that failed to come up in the conversation but which nevertheless deserved attention. The interviews ranged in duration from half an hour (32 min) to almost 2 hr (1 hr and 42 min), with an average of 62 min.

A week after the interview, respondents were e-mailed to thank them for their cooperation, and a weblink to a victim support resource was provided.

### Analysis

The interviews were transcribed *verbatim* immediately or a few days after the interview. Identifying information about the victim (e.g., name and place of residence) or the experience (e.g., the specific place of victimization) was removed during transcription. Respondents were assigned a unique number (interview 1, 2, and so forth). The transcripts were read and coded in Word using comments and text highlights.

The study used a (reflexive) thematic analysis for “developing, analysing and interpreting patterns” involving a “systematic process of data coding to develop themes” (Braun & Clarke, 2021, p. 4). The analysis involved reading the transcripts of the interviews line by line multiple times to identify patterns and themes (familiarization). The initial coding had an open structure in which main themes related to disclosure decisions (e.g., motivations, target audience, and platform choice) and the Internet as a medium (affordances) were identified. The open structure meant not having the coding steered by definitions from previous research on the online disinhibition theory to allow for a more data-driven approach. However, it appeared that the wording in the interviews at times closely resembled concepts from previous research, such as asynchronicity and visibility. After the data were thematically coded, axial coding was used. In this phase, the different codes were compared and sorted into broader themes and subthemes. Information was sought on elements that shaped disclosure decisions (e.g., target audience), informed by digital affordances (e.g., visibility). Themes regarding the pros and cons of online disclosure were analyzed and were subsequently considered in the context of the online disinhibition theory. All transcripts were revisited to determine the applicability of the codes and to select exemplary quotes for each theme to illustrate the findings and ensure the authenticity of the data. Similarities, discrepancies, possible overlaps, and exceptions were analyzed and noted in a cyclical coding process.

### Researcher Positionality

The stigmatized character of sexual violence victimization necessitates a reflection on the researcher’s positionality and participant–researcher relationship. The researcher is a white, cis-gender woman trained to conduct qualitative in-depth interviews with research experience in the field of sexual victimization, gender studies, and online disclosure. A trauma-informed approach (Campbell et al., 2019) was used. Participants’ stories were met without judgment or blame. The safety and privacy of respondents and voluntary nature of participation were priorities and discussed extensively both before, during, and after the interview. Referrals to support organizations were made available to all individuals who were contacted about the study.

## Results

The interviews revealed several online affordances of the Internet that offer opportunities and constraints when disclosing sexual violence victimization online. The opportunities generally concerned benefits of the Internet and largely related to the preliminary phase of disclosure, involving decisions about why and how experiences were shared. The constraints, or even risks of the Internet emerged mostly when the aftermath of online disclosure was discussed.

### Opportunities Afforded by the Internet

Three online affordances of the Internet were found that offer opportunities for online disclosure of sexual violence victimization, namely (a) visibility management, (b) asynchronicity, and (c) connectivity.

#### Visibility Management

The first theme that was constructed is visibility management. This can be differentiated into anonymity (managing visibility of self to others) and visibility (managing visibility of content to others and content of others to self).

##### Anonymity

Anonymity can be seen as limiting the visibility of the self to others. Respondents reflected on how the Internet offers different affordances for managing anonymity. Visibility of the self can be manipulated by using a pseudonym (e.g., on Twitter and Instagram). On other platforms, this affordance was limited for example by the “real-name policy” on Facebook, although some described how this can be circumvented.

That’s kind of the downside of Facebook, that they practically demand you use your real name. A peer said to me “gosh I’ve been kicked off of Facebook and now I have to do it using my real name”. Well, she had a service dog she absolutely loved. So, we came up with “the dog” as her last name and her real first name. (Interview 19)

Although anonymity can be seen as an opportunity, most respondents (*N* = 18, 78.3%) did not disclose anonymously and chose to share their experiences online using their own names. Reasons respondents gave for non-anonymous disclosures ranged from needing openness after years of silence; the idea that their post and message would resonate better when shared from an identified account; and the notion that sharing unrecognizably is presumed to maintain the taboo around sexual violence. “I think that the taboo should somehow be broken. If you share unrecognizably you confirm that image” (Interview 15). When asked whether the respondents had ever considered sharing their stories anonymously, the following answers were given: “No never. No. I think it is important in such a statement that someone shows their face. I think then it will resonate better” (Interview 11) and “I have already been anonymous enough” (Interview 12). One of the respondents explained feeling strength in daring to stand up for oneself; sharing the story, their name and face. “I felt strength lies in saying to the world ‘my name is [NAME] and I was abused by my father’. Not ‘I was abused by my father but you can’t know who I am’” (Interview 16).

However, a part of the respondents (*N* = 5) started out disclosing their experiences with sexual violence using a pseudonym, often out of fear of being recognized and/or consequences in the offline world. They feared ramifications for themselves and their families if they disclosed using an identifiable account. In anticipation of potential reactions, sharing anonymously felt safer.

Because I had a family and I was really afraid they would come looking for me. They would have done that at the time . . . I could have put my family, my husband and child in danger. So yes, I did want to go public, but it was safer using an anonymous name. (Interview 9)

Over time, these respondents started to disclose their names. They indicated that disclosing what had happened to them (even while using a pseudonym) increased their confidence and made them feel more resilient and capable of coping with potential consequences.

I used to do that [anonymous disclosure], when I started talking about my incest past. That’s how I once began on Facebook. A picture from the side or no picture at all. Over the years I’ve revealed myself more and more. That has to do also with my own development. I used to prefer not to have all eyes on me, but over the course of the last few years I have made myself more and more visible. Getting stronger as a result. More self-confidence, getting stronger, no longer being afraid of other people’s reactions. . . This is who I am, this is me. Period. (Interview 22)

Thus, the degree of anonymity during online disclosure seems to some extent related to the disclosure process, with anonymity decreasing in importance along the line. One of the respondents mentioned feeling as if their identity had been distorted as a result of the sexual abuse. Creating a construct online in which the best aspects of their identity were highlighted and choosing a pseudonym was considered the first step in the healing process and formation of a new identity: “Choosing a name for yourself, a construct, is already healing” (Interview 14). The respondent considered the formation of an online identity a form of acceptance, being allowed to be oneself. Online disclosure can thus, at least for some respondents, be considered as a process toward more openness related to recovery and personal growth. Disclosing more openly in turn also increased self-confidence for several respondents.

I felt myself becoming more and more secure and then at a certain point, I indeed decided to use my name. (Interview 19)

That’s how I started on Facebook. A photo from the side or no photo at all. But over the years I have come out more and more. . . That also has to do with my development. Because earlier, I was someone who preferred, uh, to stay in the background and, especially not “all eyes on me”. In the course of at least the last few years, I have come out more and more. And got stronger because of that. (Interview 22)

Finally, there is also a small portion of respondents (*N* = 5) who did not progress toward more openness and chose to disclose using a pseudonym. The anonymity provided them with the necessary feelings of safety and security. The reasons they named for wishing to remain anonymous were not wanting people close to them (e.g., family, friends, employers, and neighbors) to know about their experience and feelings of vulnerability online. The affordance to disclose anonymously gave them a sense of control over what they shared with whom.

I like using a nickname, it feels safer. . . No need for acquaintances from my hobby to know these things, like that about therapy, about me, and vague acquaintances, etc. For example, if I can ever go back to work, I don’t want my future employer to know everything about me. I always think privacy on the Internet does not exist, even if you have a closed account. It has nothing to do with secrecy. (Interview 7)

I was especially afraid that my loved ones would find out how I am doing. A lot of people don’t know that I have symptoms of depression, autism, PTSD, or bulimia. Or that I have self-mutilated. It is a conscious choice to keep it that way. (Interview 8)

Strikingly, one of the respondents used a pseudonym to disclose but did post identifiable pictures and shared the story in a video on YouTube. It is debatable to what extent this can be considered an anonymous disclosure or whether the concept of anonymity should be broadened. The respondent stated that they ensure that the surroundings in the photos cannot be traced back to the actual location so that the perpetrators are unable to find their place of residence. “I’m scared to death that someone will find my house. If they find my phone number they can call me, I can change my number. Not the end of the world. But if they find my address I will have to move” (Interview 20). The respondent wishes to be recognized by showing their face, aiming to convey their humanity. They believe that by doing so, others will be more likely to believe their stories. “I thought if I show myself then I am more likely to have people believe my story than if I just write it down. . . I think that anonymity can instill some disbelief in people” (Interview 20). However, the respondent still felt unsafe while disclosing and opted to share mainly in English to counter the vulnerability that was experienced when disclosing in their mother tongue. This example illustrates a trade-off between the wish to be validated and believed and anonymity, in which the Internet offers an alternative. Anonymity and visibility of identity are variable. Through manipulation of social media features, the examples above show it can be both increased, decreased, and maintained.

##### Visibility

Anonymity is not the only way to keep control over visibility. A second advantage of the Internet that was mentioned concerning online disclosure is control over where, when, and with whom the experience is shared. This can be considered as the second strategy of visibility management, namely *limiting* the visibility of online content. By being able to block people or only share things with certain people or on certain platforms (e.g., setting an account to private on Instagram and YouTube), respondents felt a great deal of power. Apart from constraining visibility, they can also choose to *increase* the visibility and reach of their messages. Respondents mentioned using specific hashtags (e.g., on Twitter, Facebook, and Instagram) or tagging people in their posts (e.g., on Twitter, Facebook, and Instagram).

I feel that you have a very big reach. How many people I reach, I don’t know. Someone who lives in Spain also follows me. The incest bubble that’s just people responding to each other who have gone through the same thing. That’s why I also try to use other hashtags because then my message also reaches very different audiences. (Interview 22)

The level of control individuals have over these choices and the options available depend on the affordances of the online platforms used to disclose. Digital features of a specific platform thus shape the attractiveness and usefulness of online disclosure depending on personal preferences.

Several respondents indicated using different online platforms for different purposes. For example, using Facebook to share personal details and their more publicly visible Twitter account for general awareness raising about sexual violence. For example: “Twitter I use to break the taboo of incest sexual abuse. Facebook I also use for that purpose, but there I have made a distinction between private and public posts” (Interview 22). The purpose of the specific use seems to be related to the characteristics of the platforms. The most important feature was the ability to control and regulate the degree of privacy during disclosure. For personal messages, respondents indicated they preferred to use more private platforms such as Facebook and Instagram or controlled their audience by setting filters and blocking specific users. The motive to disclose online is thus also indicative of the platform choice.

Well, there are some people I’d rather not see or don’t want them to see me. So, I have blocked them. . . Because of the sexual abuse. . . Yes, I did think about that. I have already adjusted the privacy settings on Facebook, but that was before. So that I only have people on there that I know and who know me. On Twitter, I have blocked specific people and that’s it. (Interview 4)

In addition to the possibility of sharing messages with friends only, Facebook also allows for lengthier disclosures. Despite Twitter not limiting its users in the frequency of sharing and style of writing, some respondents experienced being restricted by a maximum of 280 characters. Shorter messages leave less room for nuance, clarification, or substantiation, whereas on platforms such as Facebook, Instagram, and blogs, respondents were able to share their full stories without restrictions.

Not only did respondents use different online platforms for different purposes, but some also had different accounts on the same platform for different purposes. One respondent mentioned having a separate account for posting about sexual violence so as not to upset or trigger people close to them with shocking content. This also worked the other way around as a way of protecting themselves from being confronted with content about sexual victimization. It can be seen as a way of setting apart a place for sexual violence and a place for everyday interactions, compartmentalizing the self as a victim and the self as a social actor.

Protecting readers of disclosure messages was important for other respondents as well. For example, respondent 21 explained that they only shared details of the sexual violence experience in places where people consciously choose to read the story. The experiences were shared in varying degrees of detail on different platforms.

Yes, I often leave that [details] out. Because I find that it can sometimes come across as shocking and I don’t think you should share it. That is my limit too. . . I have shared my story on my blog and if they [readers] want to know more I refer them there too. If you really want to know, go read that. Then I don’t have to throw it on Twitter, I send the link and go read it. . . On a blog, you consciously choose to read that. Yes, they do. On Twitter, it’s rammed down your throat. . . So, when I give that link, the person still has the choice to read it or not. (Interview 21)

The above is primarily concerned with limiting the visibility of content and manipulating the affordances of the Internet to accomplish that. Some respondents also strived to increase visibility. For example, a number of respondents experienced a greater online reach on Twitter, and thus, more people were able to read their messages. The possibilities of connecting with people and starting a conversation digitally can help spread the message further. With various Twitter campaigns, many people can be reached with just a few clicks of the mouse from the comfort of their own homes. One can actively look for ways to reach the general public. One of the respondents indicated being aware of reaching the same people each time (a potential echo chamber; Barberá et al., 2015) and, therefore, used certain hashtags to reach more people.

I am actually now more aware of what I want to type, where can I put a hashtag because that then comes into that group and that is actually what I am working on now. To increase the reach because that depends on what hashtags you use. (Interview 22)

Another respondent indicated sharing in English to reach more people with their content and message. The content of the disclosures was adapted to the target audience.

I feel a lot more vulnerable in Dutch. The Netherlands is such a small country that if someone wants to find me or they somehow find my address they can be at my house within three hours no matter where they live in the Netherlands. By posting in English and making videos in English I felt that risk was less. . . [Also] I want to reach more people. If you look at the world, a very small part speaks Dutch. If I had spoken Chinese I would have done it in Chinese but I don’t so I do it in English. (Interview 20)

#### Asynchronicity

Besides having control over *how* and *to whom* specific information is disclosed and the level of visibility during disclosure, the (physical) distance created by the Internet between writers and readers was also mentioned as an advantage. The distance the respondents experienced leads to a delay in interaction with the readers of the posts—if interaction even occurs. When asked about what the Internet has to offer compared to the offline world, one respondent explained:

Being able to confront each other via the Internet, but not having to engage in conversation. If you sit across from each other, you’ll have to talk to each other. I certainly didn’t feel the need then and I still don’t. So that I could just say things without any consequences. (Interview 4)

When something was disclosed online, especially if it was intimate or personal, the respondent did not have to deal directly (or at all) with the responses of others. For some respondents, the simple act of shutting down the computer or closing the message meant it was no longer there. They were able to choose if and when they wrote messages and read responses. This distance created by the Internet was also experienced as valuable while reading disclosures from others.

That you don’t write in someone’s face. That it’s just a distance thing. Because it’s [the victimization] very, so close you know . . . these things do affect you very, very intimately and very much. That distance can help. (Interview 15)

You know, the thing is, I want to be prepared when I start talking about it and on the Internet that’s very easy. If it’s on my mind and I want to write something, I can write it and then you can read the feedback later when you’re ready. (Interview 9)

The option to write, rewrite, and even omit to post the story in the form of unlimited composition time was experienced as a great advantage of the Internet. Respondents valued being able to take the time to carefully construct their messages and prepare themselves before posting (or not posting). This again reflects a combination of control and asynchronicity afforded by the Internet. The following quotes from interviews 4 and 20 are illustrative of this: “I am fairly cautious. I write a message and then I don’t post it online or I delete it again” (Interview 4); “I think that the Internet is easier for me, is better for me. You can think before you speak. Before you press enter, you can rewrite it three times” (Interview 20).

#### Connectivity

A third affordance of the Internet for online disclosure that was considered an opportunity was the considerable value respondents experienced being able to get in touch with peers and/or find people to disclose to through connectivity. Community formation takes place on both public, private, or hidden platforms. Several respondents indicated that it is easier to find people online to share the experience(s) with than in the offline world. “Although I like face-to-face contact more, it’s harder to find people you can share with. Therefore, online is sometimes easier or nicer” (Interview 3). In addition, the Internet offers a rich source of information. Individuals can search online for information about their experiences with sexual violence, find resources for help and find platforms to disclose. The abundance of online stories reinforces feelings of not being alone and recognition.

[Online I] got a lot of recognition and that’s nice. The great thing about peers, one has progressed further than me and then you can see that it can get better. Someone else is still at the early stages and then you can see that you’ve made steps. (Interview 7)

For some respondents, disclosing online led to offline friendships with people they had met online. The connectivity and interaction with others were considered a valuable addition. Readers can provide feedback, share tips from their own experiences, or simply show their identification with the story. This way, sharing can, although for some a huge step on its own, be considered a stepping stone to sharing in the offline world.

A sense of connection. Something you generally miss in society because you can’t just talk about psychological problems. You can talk about it with people, but online. . . Plus, it’s an intermediate step from online to the real world. Because then you actually get to meet the people. (Interview 8)

### Constraints of the Internet

Disclosing experiences with sexual violence online also comes with potential constraints and even dangers. Several affordances are inherent in the use of the Internet in general, but also platform-specific features play a role. The subsequent themes were mentioned as constraints of the Internet: (a) lack of non-verbal communication, (b) disclosing online is irreversible, and (c) the Internet as a source of triggers.

#### Lack of Non-Verbal Communication

Non-verbal communication is often missing in an online environment and was described as a constraint of the Internet when disclosing online. A respondent indicated that the lack of non-verbal communication cannot be fully replaced by the use of emoticons, images, or punctuation. This lack can cause words to come across in the wrong way or posts to be misread. It was mentioned how this constraint further permeates through platforms limitation on the maximum number of characters one can use, for example, on Twitter. This can create serious challenges for online disclosure.

You have to formulate it [the story] very well for it to come across because otherwise, responses don’t make sense because then you think, “I didn’t mean it that way”. That’s the disadvantage also of WhatsApp for example. That’s that you don’t see a face, you don’t see emotions. You type something and another person can interpret it completely differently than you intended. (Interview 22)

#### Disclosing Online Is Irreversible

A second disadvantage of the Internet is that online sharing cannot be undone. Respondents were aware that once posted, disclosures are online forever, even after removal and being taken offline. A number of respondents had experienced losing control over their stories after disclosing because their posts were shared in other places, re-uploaded by other people after being taken offline, or others had stolen parts of their stories and shared them as if they were their own experiences. Respondent 11 reflected on the impact of their online disclosure going viral.

Everybody wants something from you. Not just your friends and family want to hear from you and know how you are doing, but also, holy shit, the media. I would really call it media violence because they just need a story. Everybody wants to talk to you, I got six hundred calls a day. That’s really tough. (Interview 11)

Online sharing carries the risk that stories will be taken out of context or a potential loss of the earlier mentioned control over where and with whom which parts of the story are shared.

Be aware that it’s online forever, that everyone can see YouTube, you can’t really block anyone. Because I mean if I block you tonight then you can just get another account or no account, then you can still watch, so you can’t block anyone. Everyone can see your videos forever. (Interview 20)

#### Internet as a Source of Triggers

Furthermore, the Internet presents a third constraint for individuals who have experienced sexual violence. Many mentioned how while the Internet can be a great source of information, it is also a source of potential triggers.^1^

Social media comes with a lot of ambiguity because some people post triggering photos and messages that can serve as an example for others. Naked pictures, or pictures of scars or suicide attempts. On the one hand, the recognition and the acknowledgment are nice. On the other hand, it can also be upsetting. (Interview 8)

Not all online stories of sexual victimization are accompanied by a trigger warning, and not all victims are cautious about sharing (explicit) details. For traumatized individuals, respondents explained, the Internet is filled with potential unexpected triggers. Some stated having experiences of being triggered and feeling emotional while thoughtlessly scrolling through their social media.

As you scroll through your timeline on Facebook, you don’t always want to encounter a post from someone saying “I was raped”. Sometimes you’re just on Facebook because you don’t want to do anything. To see cute cat pictures. (Interview 20)

Although it was described how being emotional or triggered was not necessarily a bad thing, being unprepared for potential disturbance while using the Internet, in addition to being traumatized, can be very upsetting. Respondents mentioned making use of a trigger warning to prevent this from happening to others.

A trigger is not necessarily wrong. In fact, a trigger is an invitation to heal. But, the important thing is to measure it in dose. So, the moment you don’t feel good about yourself, don’t continue reading. (Interview 14)

It was explained how a trigger warning indicates that the post may contain details that can trigger an emotional response. This way, readers are given a choice to either read it or not and are informed about what to expect if they decide to continue reading. The Internet, as a source of triggers, did not seem to directly drive the decision between whether or not to disclose but rather influences decisions around the content of online disclosures and reading the disclosures of others.

To me, the purpose of a trigger warning is for someone to know what they are going to read about. And then I can choose whether I want to read this now or not. (Interview 20)

On top of the potential of the Internet to trigger emotional responses, respondents mentioned addictive characteristics of the Internet. Constantly checking the number of likes or responses to their posts and feeling a fear of missing out or not belonging. This even seems to undermine the previously mentioned advantage of asynchronicity. Individuals have the option of shutting down the computer after posting, but at the same time, feel the urge to participate. It was expressed how these fears can have a large impact on one’s daily life and ignite psychological distress in itself.

The Internet is of course a fast world. On the one hand, you want to belong. If you stop, it is like you don’t belong anymore. . . I am in a lot of online groups so there is always something to see, always something to report. . . For example, yesterday I spent almost the whole day on my phone until I was really sick of it. (Interview 13)

The abundance of stories about personal experiences with sexual violence online, together with the addictive effect of the Internet and feeling a sense of responsibility for the well-being of peers, means that there is a risk of being overwhelmed by abuse stories. Several respondents indicated that at a certain point, they lost sight of reality and felt stuck in negative experiences. This can cause individuals to be dragged down constantly, instead of being helped by stories of others, until at a certain point, they feel their lives seem to consist only of abuse.

You asked about the disadvantages of sharing all of this on social media. If we think of the entire Internet as social media for a moment then, there actually have been some. It created an overload of shocking stories, which sometimes made me lose sight of reality. At a certain point, my whole life seemed to consist only of abuse, and of course, that should not have happened. (Interview 15)

## Discussion

This study aimed to gain insight into the lived experiences of victims’ online disclosure process of sexual violence and the online affordances that offer both opportunities and constraints. The disclosure process is well-studied for offline disclosure; however, the digital context differs from the offline world; communication is asynchronous and possibly anonymous and the recipient of a disclosure can be anyone. The findings were placed against the central propositions of the online disinhibition theory, serving as a conceptual framework. The focus was not so much on the decision of whether or not experiences are disclosed—respondents had also shared their stories offline—but on the decisions regarding disclosure in an online setting. This study found that online disclosure of sexual victimization entails a weighing of disclosure decisions against digital affordances and victim’s leverage of these opportunities for positive outcomes. Victims describe a reflective process with several opportunities offered by the Internet when it comes to disclosing experiences of sexual victimization online. Safety on online platforms was negotiated by being selective about where and how specific information is disclosed. There is power in having control over the content and visibility of disclosure messages as well as over an online identity.

Contrary to what is assumed in the online disinhibition theory, anonymity in itself does not seem to play an important disinhibiting role in the decision process of online disclosure of sexual victimization. A large group of respondents indicated that they had chosen not to disclose anonymously and (eventually) used their names. Some disclosed online multiple times with varying degrees of anonymity. This involved a process toward more identity revelation in which an anonymous disclosure was a stepping stone. Choices regarding identity revelation are not static. Anonymity as an added value of the Internet, in many studies referred to as the fulcrum of the online disinhibition theory (Suler, 2004), does not seem to apply to every victim. Anonymity mainly relates to *how* victims disclose, rather than *if* they disclose online. Moreover, it became apparent from the interviews that anonymity can take different forms and have different meanings—for example, disclosing in a different language or using a pseudonym but still sharing recognizable photos and videos—adding a refinement to the online disinhibition theory. Thus, despite the similar description of the themes in this study and elements of the online disinhibition theory, the meaning of items such as anonymity was found to be broader and more nuanced.

Different trade-offs were made between safety and the need to be seen, believed, and validated. The need to disclose was present, and for some victims, the possibility of disclosing anonymously works disinhibiting and offers the safety needed to tell their stories. For others, safety concerns do not seem to be a barrier to disclose online or the need for validation is greater than the fear. This is consistent with the findings of Andalibi et al. (2016). They found that victims of sexual abuse chose to identify themselves if this increased the likelihood of them being perceived as credible and trustworthy. Additionally, not being anonymous can tie individuals to collective online action in a powerful way and, therefore, be considered an advantage of the Internet (Andalibi et al., 2016). Anonymity can be maintained, increased, and decreased by manipulating the settings of social media platforms. This is reflected in the term visibility management, which is preferred over anonymity.

The visibility of the content of disclosure messages and the visibility of the self can also both be restricted and magnified by blocking certain users, using hashtags, the language of the message, and the choice of platform. Different social media platforms offer different functionalities and are also used for different purposes. Several respondents used multiple platforms at the same time to disclose their victimization. In the public part of the Internet, it is thus possible to detect different degrees of public visibility, which can be manipulated within a platform by creating different accounts. This allows for the creation of a place to share about sexual victimization that is separate from everyday interactions. Nevertheless, the platform and its set-up and architecture do not seem to be the most important elements, and victims also find a way around the restrictions of the platform, for instance, by creating a separate profile or circumventing Facebook’s real-name policy. Instead of dissociative anonymity (Suler, 2004), dissociative visibility appears to be the norm. Studying more general self-disclosure on Facebook, Vitak and Kim (2014) found similar results in which separate disclosure audiences are created to control privacy and risks. Control, asynchronicity, visibility management, and connectivity contribute to the positive assessment of the Internet for online disclosure. Like offline disclosure of sexual violence victimization, online sharing is not necessarily a one and done process (Ahrens et al., 2007). As mentioned, decisions around identity and content revelation reflect a rather fluid process in which re-evaluation of previously made choices may lead to different outcomes in the future. By asking victims themselves about these processes, it was possible to gain insight into the various options available to victims and how they could manage the features offered by the Internet at different times. At first sight, it appears to be a personal, individual decision. When considering more general patterns, a process toward more openness becomes visible that seems to be related to an individual’s recovery, which the affordances of the Internet and specific social media platforms can facilitate, for example, through using pseudonyms and removing geotags.

This study also described the affordances the Internet victims experienced as constraining disclosure, namely a lack of non-verbal communication, the irrevocability of disclosing online, and the Internet as a source of (potential) triggers. The constraints seem to be interrelated to the beneficial affordances, as two sides of the same coin. For example, the physical distance between the author of a disclosure message and the reader automatically means an absence of non-verbal communication. On the one hand, the Internet offers space to disclose without having to instantly deal with responses or consequences. On the other, the absence of non-verbal communication during disclosure can cause misinterpretations and nuances to be missed that cannot be clarified instantly.

Moreover, viral movements such as #MeToo encourage victims to publicly disclose. Observing others disclose their victimization can have a disinhibiting effect due to a perceived temporal reduction of stigma (Andalibi & Forte, 2018). Although these reciprocal disclosures contribute to awareness raising of the issue of sexual violence, victims might not consider the irrevocability of disclosing online (Gueta et al., 2020). Once information is shared online, the discloser loses at least part of the control. The loss of control is inherent to disclosure in general (Petronio, 2002), spoken words cannot be unsaid, but in the online context where information rarely ever disappears entirely and may be visible to large audiences, it causes potential additional risk for victims of sexual violence.

Asynchronicity is also at odds with one of the identified constraints, namely, experiencing an urge to monitor responses to messages and participate online. Habitual checking is a well-known characteristic of social media and can be described as “the learned sequences of automatic and routinized activation of social media checking behaviour” (Du et al., 2019, p. 477). Despite intentions to shut down the computer after disclosing, social media notifications interrupt one’s activities and even create pressure to respond (Hofmann et al., 2017). Several respondents reported feeling overwhelmed by the innumerable stories of sexual assault on the Internet, which can have a damaging effect on the individual (Mendes et al., 2018). The safety and control victims perceive as a result of asynchronicity may therefore be misleading.

The various facets of Suler’s (2004) online disinhibition theory, in research commonly presumed to have a facilitating impact on disclosure, appeared insufficiently compatible with practice. The theory does not address where, when, and how online disclosure occurs. Furthermore, the theory also lacks a reflection on the affordances of the Internet that constrain disclosure. Using the online disinhibition theory as a lens to study online disclosure of sexual violence, therefore, presents obstacles that result in an unsuitable framework. Research seems to benefit more from an affordance approach rather than using online disinhibition theory to explain decisions regarding online disclosure of sexual violence. Such an approach provides a framework for differentiating between how different affordances—both actual and perceived affordances as well as disclosure facilitating and hampering affordances—provided by the Internet can be manipulated to meet the needs of victims. The concept of affordances, as it was applied in this study, does not reflect features of the Internet or an individual but instead involves the dynamic interaction between the two. In other words, an affordance approach involves the interconnection between users and how these users apply the features of the Internet (Chemero et al., 2003). Victims’ rationalizations before a choice and effects of previously made choices, for example, expectations of what might happen after disclosure or anticipated reaction one might receive, affect the affordance trade-off. An alternative theory that fits the findings of this study does not (yet) exist. The results do suggest several considerations for theory development. First, the understanding of anonymity should be broadened and also include *perceived* anonymity. Furthermore, the term visibility management is preferred over anonymity, as visibility of the self (and the content) can be restricted as well as increased. Furthermore, to arrive at theory and predictions, both the facilitating affordances of the Internet and the constraints must be considered in relative weight of each other while taking into account victims’ previous experiences with disclosure.

The qualitative research design of this study can be considered an asset as it provides insight into the lived experiences of victims and the ways they utilize the affordances of the Internet. In doing so, this study partially substantiated, refuted, and nuanced earlier (anecdotal) evidence of the role of technology and, specifically, anonymity. Victims manipulate anonymity and visibility and learn through trial and error. We learn that victims use multiple platforms or multiple accounts on the same platform, indicating that research focusing on a single platform or affordance paints a limited view of what online disclosure entails. Disclosing sexual victimization online is not a one-off event but a process in which earlier experiences impact future choices. The phenomenon is thus broader than is visible in particular hashtag movements, something to which more attention should be paid in the future.

### Limitations

In interpreting the results, some limitations of this study should be kept in mind. First, the participant group was rather homogenous, with women being overrepresented. The experiences of victims who identify as male or nonbinary are only portrayed to a limited extent. They might, however, experience additional stigma—for example, men are less likely to disclose their victimization to anyone compared to women (Ullman, 2023)—and value affordances related to visibility management differently from victims who identify as female. To improve the wider applicability of the results, it is first of all important to examine more closely demographic factors such as ethnicity, socioeconomic factors, and gender, which impact online disclosure. Not all victims are being heard and validated after disclosing sexual violence victimization online (Salter, 2013). Future research could investigate whether groups that endure additional stigma weigh online affordances differently.

Further to the homogeneous group of respondents, a possible selection bias in the participant group should also be considered. Victims who were motivated to participate in research potentially form a specific group who are not representative of all victims who share their experiences online. Conceivably, respondents were victims with overall more positive experiences with online disclosure, some of whom had disclosed online on multiple occasions or who had processed the trauma to a certain extent. The most common reason for not participating in the study was that health problems prevented them from taking part in the research. Though speculative, it is possible that (mental) health problems were related to victimization, which may indicate a certain vulnerability. These victims possibly make choices where safety and protection are more prominent. This may, however, rather be indicative of their coping process than having to do with the characteristics of these victims. In other words, they do not necessarily form a different group of victims. A related matter was the possibility of retrospective recall bias. In the interviews, respondents were asked about reflections and decisions that had been made in the past, sometimes many years ago. In doing so, considerations of past times were interpreted from the present perspective. Retrospective recall bias may influence the recall of the decision process and the resulting choices of online disclosing and, therefore, impact the results of this study. Certain circumstances or events that contributed to the decisions may have been of great significance at the time but which the victim cannot now recall. Future research could make use of a timeline approach, whereby a timeline of events can be created to bring chronology into the story. However, the risk of inaccurate recall will always remain. A final remark is that this study only reports on decisions that resulted in online disclosure. The ultimate decision to disclose or not to disclose online is thus disregarded. Future research should also consider the decision process of victims who decide not to share their experiences online.

This study reports on the lived experiences of victims who have shared their experiences online and the affordances of the Internet that offered both opportunities and constraints. However, the role of the recipients of disclosure and the effect of their feedback should not be underestimated. From the offline context, it is known that the response of the—generally visible and familiar—audience can determine the outcome of a disclosure and if the victim is likely to disclose again in the future (Ahrens et al., 2007; Ullman, 2011; Ullman & Peter-Hagene, 2014). As the online audience is unknown, or at least invisible, the need to hide one’s identity or content may be greater, due to fear of negative repercussions. In contrast, the audience can also play a central role in ensuring that the victim feels seen and validated and that the message will be spread. The recipient of a disclosure (the audience)—or as Moors and Webber (2012) refer to it as the dance partner in an online disclosure—and the feedback (positive, negative, or neutral) they might have provided that may have factored into decisions related to, for example, identity revelation. This reflects the viral justice theory (Thompson et al., 2016), which focuses on the interaction between the victim and the audience. The public can help meet the needs of the victim; the victim can help the public to change or reinforce social and political norms. In future research, the considerations victims make and the affordances of the Internet they consider as providing opportunities or constraints when disclosing might need to be considered from a social rather than an individual perspective.

### Implications

The results of this study have implications for victims, clinicians, and support providers guiding the disclosure process. Matching the needs of victims, an appropriate medium for online disclosure can be found while at the same time helping to navigate the potential pitfalls of the Internet. Careful consideration is essential given the irrevocable nature of the Internet in inherent loss of control after disclosure. Disclosing online is also not without risk. Negative backlash in the form of misogyny, rejection, or even online hate might further inform decisions to disclose online or even have an effect on the way victims use the Internet in general (e.g., Mendes et al., 2018). Based on an individual’s wishes and expectations, an informed decision can be made regarding the platform choice and (privacy) settings. The findings also suggest a potential for informal peer support. The visibility and irrevocability of online disclosures can also provide a source for individuals who have not revealed themselves as victims, allowing them to find validation in the stories of others and opportunities to connect to peers.

This study found victims experience a spectrum of public and private online disclosure on the public part of the Internet. During the interviews, respondents also hinted at having disclosed their experiences in more secluded settings, such as private online groups on Facebook or online peer support websites. Thus far, research has only focused on online disclosure of sexual victimization in the publicly accessible part of the Web. As this study found that sharing sexual victimization on the Internet is a process toward greater openness, it is worth looking at the role of anonymity and visibility in more private online settings. Future research would do well to pay attention to this and make a comparison with disclosures in the public part of the Internet.

## Notes

1. A trigger was explained as a (negative) emotional reaction to an online post.
